# Optimising surgical management of elderly cancer patients

**DOI:** 10.1186/1477-7819-3-17

**Published:** 2005-03-23

**Authors:** Hodigere Sripathy Jois Ramesh, Daniel Pope, Roberto Gennari, Riccardo A Audisio

**Affiliations:** 1 Department of Surgery, Whiston Hospital, Prescot – UK; 2 University of Liverpool, Liverpool UK; 3 European Institute of Oncology, Milan, Italy; 4 Dept. of Surgery, Whiston Hospital, Prescot, UK & University of Liverpool, Liverpool, UK

## Abstract

**Background:**

Elderly population is on rise. It is an ethical dilemma how aggressive one should be when it comes to treat cancer in elderly. Presumed fear of increased postoperative morbidity and mortality has resulted in delivery of sub-optimal cancer surgery.

**Methods:**

In this review article we visit physiology of the aged, tools available to assess surgical risks in oncogeriatric patients, and current practice in the management of common cancers encountered in surgical oncology, with the view of increasing awareness on optimising surgical management of senior patients with cancer. A pubmed search for cancer, surgery, elderly, was carried out.

**Results:**

Cancer is on rise with increasing age predominantly affecting breast, gastrointestinal tract and lung. Increasingly more surgeons are offering surgery to elderly cancer patient but selection bias is prevalent. Available data reflect short and long-term outcome of cancer surgery in elderly is not greatly different to that of younger patient. Declining physiological reserve along with inability to respond adequately to physiological stress are salient age related changes. **C**omprehensive **G**eriatric **A**ssessment **(CGA) **is not tested in surgical patient. There is need for a tool to define individualised operative risk. Preoperative assessment of cancer in elderly is designed to offer this information based on functional status of an individual utilising currently available tools of risk assessment.

**Conclusion:**

All elderly cancer patients should be offered optimal treatment depending on their functional status not on chronological age. Oncogeriatric patient would benefit from dedicated multidisciplinary approach. Recruitment of elderly cancer patients to more clinical trials is needed to enhance our knowledge and to offer optimum treatment to this unique subgroup.

## Background

**Figure 1:**
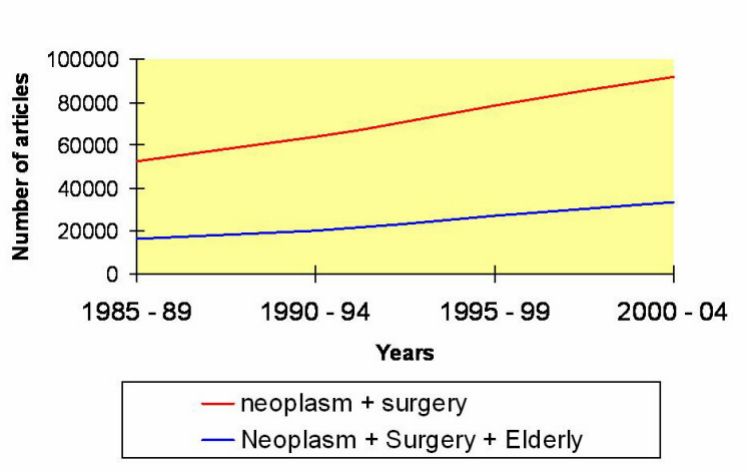
This shows the number of Pubmed articles using the search terms; neoplasm, surgery, elderly

The geriatric population is expanding, and hence the clinical decision making are often confused by effects of ageing. Age should not be the only parameter considered when addressing a medical problem [1]. There is much evidence that the booming elderly population with cancer does not receive potentially curative treatment afforded to younger cancer patient [2]. Recently there has been a rise in the number of articles published related to neoplasm, surgery and elderly [2]. Several series have proven more and more surgeons are auditing and publishing their experience in management of onco-geriatrics. This raising interest is summarised in Figure 1.

No significant difference in postoperative mortality and long term survival has been demonstrated, although a slightly higher morbidity between younger patients receiving surgical management was detected [2]. However, one might suspect a selection bias in these series. This highlights the need for prospective data collection, including co-morbidities and patient selection. Outcome of elective cancer operations in elderly could be improved by taking utmost care around peri-operative period.

In this overview we raise the concern of epidemiological changes and population dynamics, revisit physiology of aged, look into currently available instruments to assess physiology and functional status of elderly oncological patients, trends in specific organ cancers and their current treatment. Projected epidemiological data of population changes in coming years, cancer prevalence in this subset of population, and current literatures regarding management of cancers in elderly, if any, is persuasive enough to demand for a change. The priority of this issue is to increase awareness among the medical community and stimulate a debate about the urge to update the overall management of elderly oncology patient differently. Complete management of cancer in this population, and their eventual outcome, could be improved by specialist onco-geriatric multidisciplinary team. Preparing medical community to deal with this impending epidemiological time bomb is discussed.

### Epidemiological outlook

Demographic studies in industrialised countries have shown a considerable raise in the average life span and a progressive reduction in birth rates; as a result proportion of elderly patients is continuously increasing [3,4]. In developed countries in general, and England and Wales in particular, more than 15% of population is aged 65 or over 65 years [3].The demographic changes in developing countries are leading to rapid increase in the absolute number of elderly population [5].Average life expectancy in developing countries has increased from 45 years in 1950 to 64 years by 1995 and is expected to reach 72 years by 2020 [5]. In most populous developing countries namely, China, India, and Brazil, people aged 60 years and over were between 5.9 to 6.8 % in 1970 and are expected to reach 11 to 15.5% by 2020 [5]. The risk of developing cancer increases with ageing [6]. The average age of survival at birth was 40 years at the end of 19^th ^century and has now doubled to 81 years for females and 76 years for males and projected to be 86.4 years for males and 92.3 years for females by 2050 [7]. In census projection, the high life expectancy series project a US population of 416 million by 2050. In this projection 1% would be over 100 years age (4 million), 7.2 % would be over 85 (30 million), and 23.3% would be over 65 (97 million) [7], there by accounting for 31.3% of population over 65 years. Currently, average age of survival of a 70 years old woman is 15 years. A 65 years male is expected to have an active life expectancy of 12 years and 65 years female 14.09 years [8]. This expansion in life expectancy coupled with increased incidence of cancer is having a profound effect on the prevalence of cancer.

The lifetime probability of developing an invasive cancer is almost 45% in men and 38% in women [9]. The rate of death from cancer has raised from 17.7% in 1973 to 23% in 1999, while cardiac disease related death has declined from 40 % in 1973 to 30% in 1999 [9]. Very soon cancer will become the leading cause of death and more than half of new solid cancers cases occur in >70 years [9]. According to SEER data the prevalence of cancer is 207.4 cases/100,000 in <65 years old subjects and 2163.9/100,000 in >65 years in US [10]. As the incidence of cancer increases coupled with improved diagnostic certainty and life expectancy more doctors will be faced with caring for elderly patients with cancer.

Despite this epidemiological "time bomb" [11], there is concern that the scientific community has not been able to develop a significant amount of evidence-based knowledge. Only a small sub-setting of geriatric patients are being entered into clinical trials [12-16] thus elderly patients are still being managed on the basis of assumptions based on a younger population group.

### Physiological changes in elderly

The unstated fear of exposing the frail elderly patient to increased toxicities, unacceptable morbidities and high mortality rate can only be minimised and appraised by improving our insight onto the physiological aspects of geriatrics.

Ageing is defined as passage of chronological time whereas senescence is defined as the deteriorative changes with the time during post-maturation life, i.e. passage of biological time [17]. Time of onset is affected by multiple factors like diet, race, sex, physical activity, habits, hormonal effect, etc [17]. The hallmark of senescence is decreased functional reserve of individual organs and reduced ability of these organs to cope with the challenge. The progressive functional inadequacy of physiological systems is variable from species to species and individual to individual within the species [17]. It has significant impact in the peri-operative management of cancer patients, as well as the tolerance to oncological treatments, i.e. chemotherapy, radiotherapy, major curative surgery [18,37]. The organ-specific functional deterioration undoubtedly plays a significant role in the peri-operative management.

#### Cardiovascular system

Cardiac output is a product of heart rate and ejection fraction. Ejection fraction is affected by myocardial contractility and end diastolic filling. Healthy older individuals fail to increase heart rate to the same extent as younger individuals at exercise [18]. Basal cardiac output is unchanged with ageing [18,19]. Ability to increase cardiac output with ageing is more dependent on ventricular dilatation i.e., preload [19]. Aged myocardium has lowered sensitivity to beta adrenergic modulation, physiologically manifesting as lower heart rate, and lower cardiac dilatation at end diastole and end systole [18,19]. Altered pattern of Calcium regulation allows the older heart myocardium to generate force for a longer time following excitation, hence prolongs of systolic phase of cardiac cycle [20]. This in turn reduces the early diastolic filling rate by half [20]. Incomplete emptying of ventricle at end systole, hence reduction in ejection fraction is the prominent characteristics of old heart [20]. Reduced distensability, superimposed upon stressed heart could impair coronary perfusion and hence lead to cardiac ischemia [20,21].

Perioperative fluid depletion in surgical patients in general and elderly in particular is not uncommon [20]. This depletion of intra-vascular volume during peri-operative period, while undergoing physiological stress is not well tolerated by elderly [22,23]. Combined effect of depletion of intra-vascular volume, impaired response to catecholamine and increased myocardium relaxation time adversely affect the functioning of elderly patient under stress.

Cardiovascular disease increases with ageing. Cardiac arrhythmia and conduction abnormalities increase with ageing. Over half of all postoperative deaths in elderly and 11% of postoperative complications are as a result of impaired cardiac function under physiological stress [23,24]. Inexpensive objective tool i.e. supine bicycle exercise test has shown to be very useful for risk stratification for both pulmonary and cardiac complications prior to major abdominal and non cardiac surgery in ≥ 65 years [25]. Identification of this high-risk group is an aim of **P**reoperative **A**ssessment of **C**ancer in **Elderly **(**PACE**) study and their optimisation prior to surgery is the primary aim of preoperative evaluation.

#### Respiratory

Changes affecting respiratory functions are as a result of anatomical-physiological changes affecting chest wall, respiratory musculature and lung parenchyma and vasculature [26,27]. With ageing maximal voluntary ventilation, **F**orced **E**xpiratory **V**olume 1 **(FEV 1)**, **V**ital **Capacity (VC)**, decreases [26]. Reductions in blood PO_2 _Levels, decreased responsiveness to changes in blood gas levels and impaired airway protective reflexes are noted [26]. The large reserves and capacities of respiratory system allow for significant erosion in function with ageing with minimal impact on normal breathing [27]. However during increased demand e.g., exercise that age associated changes have significant impact on [26]. Reduced lung elastic recoil with increasing chest wall stiffness results in decrease in the compliance and increased functional residual volume [28,29]. With ageing impaired ciliary function in air passage and general host defence mechanisms compounded by changes in the mechanics of breathing increases the risk of atelectasis and postoperative pulmonary infections [30]. Literature quotes 9 to 18% of elderly patients suffer from respiratory complications during postoperative period after major non-cardiac and thoracic surgery [25,31]. Aim of preoperative assessment is to identify this high risk and optimise them prior to major surgery.

#### Body composition

Changes in the body weight total body water, body fat distribution and muscle mass may affect response drug therapy in general and anaesthetic drug in particular. Proportion of body fat increases with age and is accompanied by concomitant decrease in the total body water and skeletal muscle mass [32]. As the total body water is reduced there is a reduction in the volume of distribution of water soluble drugs. [1,32]. This in association with decreased renal clearance account for higher plasma levels of water soluble non-depolarising muscle relaxants in the aged than in younger patients [33,34]. Similarly increase in the total body fat affect the distribution of lipid soluble drugs and could prolong the effect [35].

#### Fluid, electrolyte and renal physiology

Disorders of fluid and electrolyte balance are very common in elderly. The homeostatic reserve decreases with ageing. By the age of 70 years half of the original nephron complement may have been lost [36]. Loss of renal cortical mass reflects in decline of renal function, decrease in the glomerular filtration and tubular function and hence inability to concentrate urine [36]. Serum creatinine may remain stable masking underlying progressive loss of renal function [37]. Philip *et al*, in 1984 demonstrated decreased thirst sensation in elderly [37]. The renal responsiveness to vasopressin is impaired, similarly renal function and renin-aldosterone decreased [37]. Elderly people under metabolic stress become dehydrated and acidotic. This compounded by reduced plasma flow and impaired handling of nephrotoxic drugs renders them susceptible for acute renal failure [38]. This emphasise the need to identify high-risk elderly individuals during preoperative and Peri-operative time to deliver optimal treatment and to assist them in smooth recovery from surgery.

#### Liver functions

An elderly patient in general starts with a 20% decrease in plasma albumin concentration. Peri-operative nutritional needs of elderly cancer patient deserve special attention for above reasons. The process of detoxification, conjugation, and extraction of different compounds seems to be remarkably preserved under normal conditions despite actual decrease in the size of the organ. However under hypermetabolic states, the liver may fail in increasing its synthetic and metabolic functions [39,40].

#### Skin and wound healing

The changes seen in aged skin are a combination of effects from intrinsic and extrinsic factor [41]. Characteristic changes seen with ageing like dermo-epidermal atrophy, dryness, roughness, sagging, and wrinkling, have implications for wound healing [42]. Wound healing in elderly presents a major clinical and economic problem [42]. Evidence of age-related wound healing has been derived from most past empirical observation [42]. The clinical impact of change in the tensile strength of wounds, rate of wound closure, and accumulation of wound healing in acute wound healing appears to be small. The consensus is that effect of ageing on wound repair is primarily a temporal delay and not an actual impairment in the wound healing. Though there is a temporal delay in healing in aged it is not any qualitatively different to that of younger [42]. Poor healing in chronic wounds more often related to comorbid conditions than age alone.

### Function Assessment

Ability to withstand the stress of various forms of treatment for cancer in elderly patient is dependent on the functional reserve and ability to build an appropriate response to the stress. A large number of instruments have been developed over the years by geriatricians, although some of them are time consuming and impractical in our busy clinical setting. Amongst the others, we found the following validated instruments particularly useful in predicting outcomes in a prospective oncogeriatric series receiving chemotherapy (table 1) [43])

**Table 1:** Validated instruments in elderly chemotherapy patients

Mini Mental Score (MMS) [44]
Activities of Daily Living (ADL) [45]
Instrumental Activities Daily Living (IADL) [46]
Geriatric Depression Scale (GDS) [47]
Brief Fatigue Inventory (BFI) [48]

No scoring method relating to candidacy for surgery has ever been attempted specifically on oncogeriatric population.

### Surgical risk in elderly

Surgeon is often called to make a decision to operate or not to operate on a patient and it is one of the most important decisions that he has to make in surgical practice. This assumes greater importance when dealing with a patient who is a poor surgical candidate. In current day practice there is a high demand for individualised risk assessment to be shared between the surgeon and patient. Risk prediction should be appreciated and disclosed to the patient at the time of consenting.

Chronological age is an unreliable predictor of performance of health in an individual. The largest numbers of abnormal laboratory tests are not capable of predicting post-treatment and operative adverse outcome [49]. Over half a century ago Welch reported in his large series of abdominal operation in-patients over 70 years of age, a peri-operative mortality of 20.7%. He concluded that surgery itself is safe but that aged required greater attention in peri-operative management [50]. Present evidence suggests that health of extreme elderly is improving and interventions can be successful at late ages. The Postoperative mortality, morbidity and long term survival after cancer surgery for solid tumours are not affected by chronological age on its own [51-53]. Currently ongoing multinational study **P**re-operative **Assessment **of **C**ancer in **E**lderly **(PACE) **is trying to provide a scoring method to assess candidacy for surgery of oncogeriatric populations [54]

Widely known American Society of anaesthesiologists (ASA) [55] physical status system is not aimed at measuring operative risk, rather it assess globally the degree of sickness or physical state prior to anaesthesia and surgery. ASA is insensitive to differentiate largest proportion of patients in ASA II and III [56]. Surgeons to assess cardiac risk in non- cardiac surgeries rarely ever use Goldman cardiac risk index **(CRI) **[57]. **A**cute **P**hysiology **A**nd **C**hronic **H**ealth **E**valuation **(APACHE) **is probably the best known of the physiological scoring systems based on 34 physiological variables taken in the first 24 hours of patient's admission. APACHE II using 12 physiological variables is well suited for intensive care unit patients needing ventilatory support [58]. In general surgical patients' not needing respiratory support its use is limited. **P**ortsmouth modification of **P**hysiological and **O**perative **S**everity **S**core for En**U**maration of **M**ortality and **M**orbidity **(P-POSSUM) **[59] a modification of POSSUM and POSSUM consists of physiological score and operative score. Latter component of POSSUM score is highly relevant to the final outcome; unfortunately necessity of per-operative variables compromises its usefulness as preoperative assessment tool.

A comprehensive geriatric assessment (CGA) based on the previous parenthesis might improve our understanding of the surgical risk, allowing a more accurate comparison of surgical series, a careful patient selection, and adequate consenting.

CGA has proven to be useful in predicting mortality and morbidity in several clinical settings including hospital geriatric evaluation, inpatient geriatric consultation, home assessment service, hospital home assessment service and outpatient assessment service and in a number of chronic diseases [60-63]. It is not a mere list of associated medical conditions that have impact on prognosis but it is actually complexity of information that can be gathered through CGA. CGA adds substantial information to the functional assessment of elderly cancer patients routinely collected through performance status **(PS) **index [60,6].

### Preoperative assessment of cancer in elderly (PACE)

An international project has been launched aimed at defining the general health condition of oncogeriatric surgical candidates. PACE is a tool designed to assess the functional activities of geriatric patient there by making an attempt to assess the functional life of an oncogeriatric patient and there by predicting the individualised risk of cancer surgery. Patients age 70 years constitute 90% of the study subjects and present signs of ageing. Patients ≥ 70 years undergoing moderate, major and major + elective surgery whose mini mental score is 18 and above and are able to give written informed consent have been included in this trial. It aims at predicting the probable outcome of cancer surgery treatment preoperatively in elderly. The tools incorporated in the PACE are detailed in table 2.

**Table 2:** Validated Instruments Used with PACE

Mini Mental State Examination
Satariano's Modified Index of Comorbidities
Activities of Daily Living
Instrumental Activities of Daily Living
Geriatric Depression Scale
Brief Fatigue Inventory
Eastern Co-operative Oncology Group Performance Status
American Society of Anesthesiologists Physical Status
Physiological and Operative Severity Score for EnUmeration of Mortality and Morbidity – (POSSUM)
Portsmouth POSSUM Modification

The surgical outcome is defined by 30 day mortality and 30 day morbidity as assessed by care delivery team. The following complications are included in 30 days morbidity checklist (table 3). Our pilot study has proven PACE is feasible, inexpensive and well accepted by the patient (table 4) [64].

**Table 3:** 30 Days Morbidity check

**Complications**	**Absent**	**Minor**	**Major**
Respiratory			
Cardiac failure			
Renal failure			
Generalized sepsis			
Stroke & Neurologic			
Haemorrhage & Bleeding			
Nutritional problems			
Other organ failure			
Wound			
Infection/Dehiscence/Fistula			
Thrombo-embolic			
Hepatic failure			
Urinary retention			
Anastomotic Failure			
Peripheral ischemia			
Endocrine failure			
Pressure Sores			
Analgesic problems			
Others			

**Table 4:** Pilot study results: Association of PACE with postoperative morbidity

**Components**	**Complications 24 patients**	**No complications 48 patients**	**P value**
Median (IQR)			
Co-morbidities	3.2 (2–5)	3 (2–4)	0.506
MMS	29 (26–30)	27 (26–29)	0.070
GDS	4 (1–7)	2 (1–4)	0.056
BFI	3 (1.1–5.8)	9 (1.3–29.5)	0.216
Number of patients (%)			
PS = 0	11 (45.8)	41 (85.4)	0.002
ADL (independent)	4 (16.7)	32 (66.6)	<0.001
IADL (dependent)	14 (58.3)	38 (79.2)	0.207
ASA = 1 or 2	17 (70.8)	37 (77.1)	0.681

Performance status was found to be significantly lower in patients who developed morbidity and lower activities of daily living was associated with higher postoperative complications. Recruitment to the study is currently ongoing and full results will be revealed on completion of this study [64].

### Solid cancers and surgical management

Surgery remains the treatment of choice for solid cancers. There is significant under representation of elderly in trials of treatment for lung, colorectal, breast and ovarian cancers [65]. In the following paragraphs, we discuss current trends in the management of common solid tumours encountered by general surgeon.

### Breast cancer

Breast cancer in elderly women is a significant health problem. Elderly women have 6-fold higher incidence and 8-fold higher mortality rate compared with non-elderly women [66]. Current incidence rate of breast cancer remaining constant, it is projected that a 72% increase in the number of elderly women diagnosed with breast cancer in US by 2025 [66]. Fifty percent of breast cancers occur after the age of 65 years and 25% after the age of 75 years [67]. The number of women diagnosed with breast cancer is likely to increase due to demographic changes. Trends in the cancer mortality for all cancers in elderly have long been unfavourable. Since late 1990s total cancer mortality between ages 65 and 84 has been declining in the European Union (EU) [68]. Breast cancer mortality has declined over the last decade by 8% in US and by 3% in the EU [68].

Our understanding about breast cancer treatment in elderly is mainly based on retrospective and observational studies and on very few randomised clinical trials [69]. Surgery is the main stay of treatment for early breast cancer independent of age and was the usual therapy for all ages till 1970s [70]. Primary tamoxifen treatment was adopted enthusiastically in 1980s with publications of promising early results with tamoxifen in patients over 70 years [71], 81% of elderly women treated with primary tamoxifen appear to develop progressive disease after 12 years of follow up as against 38% with mastectomy alone [71].

The drawback of tamoxifen only treatment was the short duration of response [72]. Patients who relapse face the prospect of second line hormonal treatment or surgery or radiotherapy [72]. A change in treatment plan in favour of surgery is welcome in view of expected active life of 14 years for a 65 years old lady. In general, surgery appears to be well tolerated despite patient age [71].

Large proportions of elderly cancer patients are offered less than conventional treatment. Elderly breast cancer women are offered breast conservative surgery but are less likely to have axillary dissection, postoperative radiation and chemotherapy [73-75]. Local recurrence rates after conservative surgery without radiotherapy are reported between 3 to 47% [76-81]. Mortality rates after breast cancer surgery in elderly is <1% [82-84]. Predominant morbidities are related to wound complications [79,83]. Fentiman *et al*, in their multicentric randomised trail investigated quality of life (QOL), survival at 12 months and treatment preferences in elderly patients (≥70 years) with early breast cancer undergoing mastectomy or tumour excision plus tamoxifen [85]. Patient undergoing tumour excision and tamoxifen did not differ from those undergoing mastectomy in terms of fatigue, emotional functioning and fear of recurrence. Conservative breast surgery patients reported fewer arm problems and a borderline shift in the direction of benefit of body image (P = 0.06) [85]. QOL was better after conservative breast surgery and such treatment is to be individualised and to be preferred independent of age [85].

Axillary surgery plays a key role in management of breast cancer surgery either to achieve nodal disease control or to stage the tumour accurately therefore to decide on adjuvant therapy. Denying axillary surgery exposes the patient to increased risk of nodal disease recurrence, on the other hand full axillary clearance increases morbidity. Although the risk of increased morbidity secondary to full axillary surgery is not higher in elderly patient [86,87], axillary surgery is less often undertaken in the elderly patient [88]. Confusion still prevails regarding optimum axillary surgery. To bring new light on this controversy Chetty *et al*., carried out a randomised clinical trial comparing regional control rate for sampling with axillary clearance. No significant difference was found. Unfortunately elderly patients were excluded from this study [89]. Morbidity related to axillary surgery in elderly was shown to be no greater than young. Further research is needed to guide us in choosing the appropriate axillary surgery for breast cancer in this age group.

Primary endocrine treatment has been substituted for surgery based on results from 1980's experience when ER status was unknown [77], 100% disease progression has been noted in ER negative group [78,90-92]. Fennessy concluded that tumour excision decreased the mortality rate in an unselected population of elderly women with operable breast cancer who were fit for the procedure [93]. With the current evidence primary endocrine therapy should be reserved for highly selected ER positive elderly breast cancer patient who are unfit to have surgery or respecting patient wishes.

Several retrospective studies have reported age alone should not determine the type of breast reconstruction. Autogenous tissue reconstruction can be a safe successful alternative for women aged 65 year and above [94]. Further research is needed to answer outcome of breast reconstruction after mastectomy for cancer in elderly.

The definition of upper age limit for breast cancer screening is a very complex issue. Screening benefit depend on life expectancy. People with < 5–10 years life expectancy are unlikely to benefit from screening, so it is worth while considering the variability of different ages. Medical system has the ethical obligation to properly inform the population invited for screening. Further research should be encouraged to answer the benefit of mass population breast screening in elderly.

The ancient assumption that breast cancer was not worth resecting in the aged population is finally rejected in view of a minute morbidity and mortality rate and obviously improved cancer outcomes for patients who received surgery. It is reassuring to know how the performance of the largest part of breast surgeons is not significantly biased by an ageist mentality. There is a need for research targeting specific needs of elderly patients with breast cancer and develop a specific treatment guideline in this group. Recent survey among breast surgeons in the UK has demonstrated the need of a standardised pre-operative assessment capable of characterising the patient's "functional age", in order to optimise treatment planning and stratify outcomes on the basis of factors other than chronological age [96].

### Melanoma

Melanoma is a significant public health problem. Incidence and mortality from cutaneous malignant melanoma continue to rise [96]. Melanoma is often diagnosed late in elderly due to multiple reasons. Many retrospective studies have predicted prognosis of melanoma as age independent [96]. Greater numbers of thick lesions are increasingly seen with rising age [97]. On the contrary, Cohen *et al*, in 1987 predicted age as an independent poor prognostic factor for death due to melanoma [98]. Literature search did not yield any prospective randomised trial to address effect of age on melanoma. With nose and ear lesions increased [98], the percentage of elderly patients with metastatic disease at initial diagnosis did not vary compared to younger individual [98]. Histotype lentigo maligna lesions were seen with increased frequency in elderly [97]. Chang *et al*., in their retrospective evaluation of intermediate thickness and T4 (≥ 4 mm) melanoma lesions in ≥ 65 years noted lymph node status is the most important prognostic factor influencing overall survival **(OS) **and disease free survival (DFS) [96,99]. Surgery plays pivotal role in treating melanoma. The size of resectional safe margins is still being debated in young and elderly alike, but there seems to be no data suggesting a differentiated approach. Results of retrospective study elderly patients with positive nodal status, who received no adjuvant treatment, did significantly worse than historical control [96,99] They concluded treatment for melanoma in ≥ 65 years should be aggressive and should not be denied adjuvant therapy based on age alone [96,99]. There is no consensus regarding optimal nodal surgery for melanoma in elderly. Prospective controlled trial is needed to provide answer to optimum nodal surgery for cutaneous melanoma in elderly.

Older patients are assumed to have higher risk of complications from isolated limb perfusion (ILP). Nooda *et al*., did not find significant difference in complete response rate, loco-regional relapse, limb toxicity, systemic toxicity, local complications and long term morbidity of ILP between < 75 years and ≥ 75 years [100]. They concluded older age is not a contraindication for ILP. It is prudent to treat melanoma in elderly with same radical approach as in younger patient without age bias [100].

The predictive value of screening diagnosis of melanoma was more than twice as high for men ≥ 50 years with either a changing mole or skin types I and II compared with other participants [100]. The yield of mass screening for melanoma could be improved by outreach to middle aged and older men [101]. Impact of formal assessment of targeted screening warrants a further study.

### Lung cancer

Lung cancer is the leading cause of cancer-related deaths in population aged over 70 years in the western world [102]. Over half of the people diagnosed with lung cancer are over 65 years old. Compiled data from 33 countries in four continents shows increase of 180 % to 580% in the mortality amongst 65 – 84 years old males and females with lung cancer respectively from 1955 to 1992 [103]. 25 to 40% of all small cell lung cancer [103] and 40% of non-small cell lung cancer (NSCLC) [105] are in ≥ 70 year. Lung cancer mortality rates have declined over last decade by 8.5% in the EU and by 0.9% in USA [105]. Elderly cancer patients are less likely to enrol (1.3%) in co-operative group than younger patients (3.0%) [106]. Surgery offers the best potential for cure in patients with carcinoma of the lung, as is the case with most solid tumours. Patients with untreated or palliated early stage NSCLC have an average life expectancy of only 1.5 years [107], while individuals in the ninth decade of life have a 50% chance of living an additional 5 to 9 more years [108]. In case of resectable primary lung neoplasms, surgery remains the treatment of choice independent of age, as is the case for most solid tumours. No significant difference in survival or cancer related survival after lobotomy Vs limited resection has been noted between elderly and young [109]. Many clinicians avoid surgery or minimise surgical procedure on the basis of age but recent advances in the preoperative risk assessment and surgical and anaesthetic techniques have resulted in a significant decrease in operative mortality and morbidity [110]. Age is a recognised risk factor for death after thoracotomy in elderly patients with lung cancer. Lung sparing procedures such as segmentectomy and wedge resection are being increasingly performed for lung cancer especially in elderly patients [111-113]. Recent advances in **V**ideo **A**ssisted **T**horacic **S**urgery **(VATS) **techniques [114], voice controlled robotic lung resections [115], provides an alternative approach to standard thoracotomy in elderly lung cancer patients resulting in decreased recovery time and fewer postoperative complications. In a multi institutional trial of patients with stage I NSCLC undergoing lobectomy by muscle sparing thoracotomy or VATS confirmed that the latter approach decreases the incidence of postoperative complications [116]. Long-term survival after a VATS lobectomy for NSCLC has been reported to be comparable to that achieved by open thoracotomy [117]. VATS lobectomy has been proven to be feasible and relatively safe alternative in-patient with poor cardiopulmonary status for early lung cancer [118]. The treatment of stage III NSCLC is still a matter of debate since the efficacy of surgery decreases and operative mortality increases with stage of disease. The reluctance to offering surgery for the elderly is particularly evident in these advanced stages that require more extensive resections. Combined modality treatments offer an improved outcome for patients with stage III lung cancer [119]. Unfortunately, elderly patients have been under represented in these trials. More studies are warranted in order to define if these conclusions can be extended to the elderly population as well. The multi-disciplinary approach to lung cancer acquires importance when treating elderly patients. The close participation between pulmonologists, oncologists, thoracic surgeons, anaesthesiologists, cardiologists, geriatricians, primary care physicians, physical therapists, and nutritionists on the pre- and postoperative course of the elderly with lung cancer can improve measurable outcomes and decrease their frustrations, therefore improving their quality of life [118].

### Oesophageal cancer

Oesophageal cancer is typically disease of aged man. Estimated annual incidence is 7.7/100,000 in European community inhabitants. In recent years, the number of elderly patients with oesophageal cancer has steadily been increasing. An incidence of 14.5% of all oesophageal cancer was seen in years 70 [120]. Our current limited knowledge regarding management of oesophageal cancer in elderly comes from several retrospective series. Peracchia *et al*., reported a consecutive series of 1338 oesophageal cancer patients of whom 18% were 70 and above, overall hospital mortality was 6% and five years actuarial survival rate was 30%. This is comparable to the survival of younger patients [121]. The diagnosis is often delayed in elderly [122]. Hence, they present with complications like malnutrition and aspiration pneumonia. Some physicians believe that aggressive surgical approach is imprudent because of alleged higher rates of mortality and morbidity and lower rates of survival than younger patient is. Ellis *et al*., while reviewing their 27 years experience from January 1970 to Jan 1997, noted a total of 505 patients had surgery for cancer of oesophagus, 29% of these were ≥ 70 years. Actuarial 5-year survival rates were 24.1% in ≥ 70 years as against 22.4% in younger patients. The in-hospital mortality though was higher in elderly group but was not statistically significant [123]. They concluded age should not be a limiting factor in using aggressive surgical approach for management of cancer of oesophagus or cardia in patients aged 70 years or older. Similarly Thomas *et al*., compared between <70 years and ≥ 70 years, operative mortality (10.7% Vs 11.2%), Major morbidity (10.7% Vs 13.6%), pulmonary complications (17.9% Vs 20.6%), and 5-year survival rate (17% Vs 18.9%) and concluded that oesophagectomy can be safely performed in septuagenarian patients [124]. No differences in surgical resection rates were noted between patients younger or older than 70 years [125]. Elderly patients (>80) had an overall higher respiratory and cardiovascular complications than younger < 70 years. The operative mortality was 4.7% for < 70 years as against 5.6% for over 80 years. 5-year survival inclusive of operative mortality was similar 25.1% in over 80 as against 19.8% in under 70 years [125]. They concluded that survival benefit is similar to that in younger age groups enforcing the view that oesophagectomy can be safely offered in specialist units with acceptable long-term survival in all ages groups. Long-term survival in oesophageal cancer is related to stage of tumour at presentation [126] (stage I = 73% Vs stage III = 6%).

There is an urgent need for targeted research and prospective trial to understand management of oesophageal cancer in elderly. This could be achieved by active recruitment of elderly oesophageal cancer patients into clinical trials.

### Gastric cancer

Cancer of the stomach is still a common cancer in Europe with an annual rate of 35.7/100.000. Its incidence peaks around the age of 70 and is uncommon before the age of 40 years [127]. In elderly, gastric cancer is more likely to affect the distal part of the stomach [127] and hence there is a higher prevalence of stenosis and anaemia. Elderly patients suffer more from a well-differentiated tumour and frequently of intestinal type [128,129]. There is some evidence that young patients with gastric cancer have worse prognosis and have a higher prevalence of diffuse type [130].

Surgery is the treatment of choice for gastric cancer independent of age. The three-year survival rates among octogenarians having gastric cancer surgery for advanced and early gastric group were superior to no surgery group [131]. The early and long-term outcomes in elderly are comparable to that of younger patient. Age alone should not preclude gastric resection in elderly gastric cancer patients [132].

Due to prevalence of respiratory and cardiovascular co-morbidity in elderly they are often placed at higher ASA classes. Maehara claims that the operative mortality has been significantly reduced in recent years. Reporting on 344 patients who had surgery for gastric cancer, he shows a drop to 0% in most recently operated patients [133]. The ratio of partial to total gastrectomy has declined to 60% Vs 40% while the ratio of R0-R1 Vs R2 – R3 has increased from 45% Vs 65% to 25% Vs 75%. Though the morbidity is slightly higher than younger age it is not statistically significant. The five-year survival does not seem to be affected by patient's age [133]

Current practice of gastric cancer treatment in elderly is based upon results from that of younger patient and from retrospective series. The long-term cancer related prognosis of elderly gastric cancer patients does not differ significantly from that of younger patients, elective surgery being feasible with standard morbidity.

### Colorectal cancer

Colorectal cancer (CRC) is the second most common cancer in US, primarily a disease of elderly (138,000 new cases / year) [134,135] More than half of deaths from colon and rectal cancer occur in 70 years and above. Its incidence increases with the increasing age. Based on the census projection colon cancer related admissions in US will increase from 192,000 to 448,000 by 2050 in people aged ≥ 60 years [136]. Median ages at diagnosis ranges between 70.5 years for colon and 68.4 for rectal cancer [136]. **A**ssociation of **C**olo**P**roctology of **G**reat **B**ritain and **I**reland **(ACPGBI) **in its recently published booklets has confirmed high prevalence of cancer in elderly [137]. It has been shown that patients in the age group of 65–74 were 1.8 times more likely to die following surgery compared with 3.5 times for 75 to 84 years and 5 times for over 85 years. These odds ratios were not, however, adjusted for all the other risk factors (ASA, site, and stage) and should be interpreted with some caution.

Right-sided carcinoma shows an increased frequency in the older patient [1]. Advanced colorectal tumours are common in elderly explaining the greater proportion of palliative surgery in this group [138]. Obstructive tumours are significantly more common in patients over 70 years of age. Elderly patients with CRC are still presenting as surgical emergencies (obstruction and/or perforation) in up to 40% of cases. Higher reported incidence of palliative surgeries being performed, and a lower overall utilisation of neo-adjuvant preoperative and adjuvant postoperative therapies; influences long-term cancer related outcome [139,140]. Elderly rectal cancer patients continued to be denied surgery i.e., 11% in ≥ 70 years Vs 1% < 70 years [141]. There is paucity of data regarding adjuvant treatment of older patients with rectal cancer and hence there is an urgent need to enrol more elderly rectal cancer patients in to trial to fully evaluate the outcomes of cancer therapy in this subpopulation [142].

First line of treatment for colorectal cancer is surgery. Total mesorectal excision (TME) has become new standard of operative treatment for rectal cancer replacing conventional receptions [143]. Quality of life and functional results after low anterior resections and TME in the elderly are of no worse than in younger patient [144], 85% of patients over 75 years, who had sphincter saving rectal surgery denied any significant problem with bowel function or continence [145]. Liver resection for colorectal cancer liver metastases in properly selected elderly patients ≥ 70 years is feasible and age alone is not a contraindication [146]. Clinico-economical evaluation of elective colorectal cancer in the aged independently analysed cost of preoperative investigations, operative real cost postoperative real costs and median total charges. The economic burden when delivering radical surgery to the aged colorectal cancer and younger ones were shown to be statistically no significant [147]. Five-year cancer specific survival disease free rates were not different between young and elderly. Postoperative morbidity and mean hospital stay were not different between young and elderly having colorectal surgery [138]. Evidence available today fails to support the practice of denial of curative colorectal cancer surgery in elderly under elective situation. There is very little rationale for substandard treatment delivery in colorectal cancer surgery, as the long-term cancer specific survival rates do not differ according to patients' age under elective conditions [1,146].

Minimal invasive surgery like laparoscopic assisted colectomy is emerging as alternative to open surgery for colonic cancer. Early reports shows that it is a viable option and some elderly patients with CRC have been treated with as part of these protocols [147]. The safety and efficacy of laparoscopy assisted colectomy remains unclear, awaiting the final results of the **C**linical **O**utcomes of **S**urgical **t**herapy **(COST) **trial designed to examine whether it is an effective alternative to open colectomy in the prevention of recurrence and cancer mortality [148].

The emergency surgery is associated with a significantly higher incidence of operative mortality at any age (15% on emergency Vs 5% Elective surgery [1,137]. No significant difference in mortality is recorded between two age groups i.e. <70 years and > 70 years when only elective operations are considered i.e. 4% and 7.4 % respectively.

Trans-anal excision of low rectal cancer in selected patients is an acceptable alternative to formal resection. The recent development of trans-anal endoscopic microsurgery **(TEMS) **has permitted removal of tumours from the upper rectum. Important selection criteria include early T stage, good or moderate differentiation, relatively small tumour size and negative microscopic margins [150] are factors important in curative resections, although criteria for deliberately palliative endorectal resections may be relaxed in selected elderly patients. Local recurrence and survival rates seem comparable to TME in early rectal cancer where TEMS is used with curative intent [151-153].

## Conclusion

Surgeons will have to deal with increasing cancers in elderly. Current treatment practices in elderly are based on experience gained from retrospective series and reviews. An attempt to transfer the results of younger patients to this heterogeneous population should be discouraged. Better understanding of our knowledge about this unique age group could be achieved by encouraging active participation in clinical trials and education of medical community. The very finely balanced physiological resources in elderly cancer patients, demands extreme care around pre- and peri-operative. The cost of treatment of cancer in elderly is no expensive than those in younger patient. The enthusiasm to offer cancer treatment to selected oncogeriatric patients in par with young, a practice prevalent among minority of medical community should hence forth not only include oncogeriatric community without selection bias but also spread to wider medical community members. A currently ongoing multinational trial (PACE) is aimed at overcoming this selection bias and assist treating physician to make informed decision about optimum treatment in discussion with patient. Oncogeriatric patients would benefit from complete care with active participation of multi-disciplinary team comprising of Surgeons with special interest in geriatric cancer surgery, Geriatricians, Geriatric oncologists, Anaesthetist with interest in geriatric anaesthesiology and social worker. Further research into tailored treatment of elderly cancer patient from the time of preoperative evaluation to and including optimum surgery for individual cancers and adjuvant therapy is needed.

### A look at the future

Improvement in care of oncogeriatric subgroup comes with better understanding of elderly cancer patients by recruiting them into specifically designed clinical trials. Changing the attitude of treating surgeons towards the elderly with cancer comes with widening our knowledge. The benefit of technological advancement should percolate to elderly to improve their quality of life aside long-term survival. The benefit of advancements in science like early detection of cancers, improvement in anaesthetic techniques, and surgical techniques, should be extended based on individual merits independent of age. It is the responsibility of today's doctors to train future generations to offer treatment without age bias but on individual merits. This could be achieved by bringing gerontology in par with paediatrics to class room teaching. Proactive participation of practising doctors and up coming surgeons in ongoing national and international educational and scientific meetings should be encouraged.

## Conflict of interest

The author(s) declare that they have no competing interests.

## Authors' contributions

• **Hodigere Sripathy Jois Ramesh**

• Study management – literature review/update & preparation of manuscript.

• **Daniel Pope**

• Statistical analysis & study design

• **Roberto Gennari**

• Study material contribution & intellectual input

• **RAA**

• Study design & management

## Funding Source

• None

## References

[B1] Audisio RA, Veronesi P, Ferrario L, Cipolla C, Andreoni B, Aapro MS (1997). Elective Surgery for gastrointestinal tumours in the elderly. Ann Oncol.

[B2] Audisio RA, Bozzetti F, Gennari R, Jaklitsch MT, Koperna T, Longo WE, Wiggers T, Zbar AP (2004). Surgical management of elderly cancer patients: recommendations of SIOG surgical task force. Eur J Cancer.

[B3] Grundy E, Raymond T, Howard F, Brocklehurst JC (1999). Epidemiology of Ageing:. Brocklehurst's Textbook of Geriatric Medicine and Gerontology.

[B4] Kinsella K, Suzman R, Robine JM, Myers G, Evans JG, Williams TF, Beattie BL, Michel JP, Wilcock GK (2000). Demography of Older populations in developed countries. Oxford Textbook of Geriatric Medicine.

[B5] Kalache A, Keller I, Evans JG, Williams TF, Beattie BL, Michel JP, Wilcock GK (2000). Population ageing in developing countries: demographic aspects. Oxford Textbook of Geriatric Medicine.

[B6] Gosney M, Raymond T, Howard F, Brocklehurst JC (1999). Geriatric Oncology:. Brocklehurst's Textbook of Geriatric Medicine and Gerontology.

[B7] Day JC (1996). Population projections of United States, by age, sex, race and Hispanic origin: 1995 to 2050. series P25 – 1130.

[B8] Redmond K, Aapro MS, Veronesi U, Aapro MS (1997). Cancer in elderly, a growing problem. Cancer in elderly ESO Scientific updates-2.

[B9] Monson K, Litvak DA, Bold RJ (2003). Surgery in the aged population: Surgical oncology. Arch Surg.

[B10] Lag R, Eisner MP, Kosary CL (1999). SEER Cancer Statistics Review 1973–1997. Cancer in the European Union (1995).

[B11] Redmond K, Aapro MS, Veronesi U, Aapro MS (1997). Cancer in elderly, a growing problem. Cancer in elderly ESO Scientific updates -2.

[B12] Masoro EJ, Masoro EJ (1997). Aging: Current concepts. Handbook of physiology: Aging.

[B13] Barchielli A, Balzi D (2000). Age at diagnosis, extent of disease and breast cancer survival: a population-based study in Florence, Italy. Tumori.

[B14] De Rijke JM, Schouten LJ, Schouten HC, Jager JJ, Koppejan AG, van den Brandt PA (1996). Age-specific differences in the diagnostics and treatment of cancer patients aged 50 years and older in the province of Limburg, The Netherlands. Ann Oncol.

[B15] De Rijke JM, Van der Putten HW, Lutgens LC, Voogd AC, Kruitwagen RF, Van Dijck JA, Schouten LJ (2002). Age-specific differences in treatment and survival of patients with cervical cancer in the Southeast of the Netherlands, 1986–1996. Eur J Cancer.

[B16] De Rijke JM, Schouten LJ, Hillen HF, Kiemeney LA, Coebergh JW, Van den Brandt PA (2000). Cancer in the very elderly Dutch population. Cancer.

[B17] Masoro EJ, Raymond T, Howard F, Brocklehurst JC (1999). Physiology of Aging. Brocklehurst's Textbook of Geriatric Medicine and Gerontology.

[B18] Lakatta EG, Gerstenblith G, Evans JG, Williams TF, Beattie BL, Michel JP, Wilcock GK (2000). Cardiovascular Disorders. Oxford Textbook of Geriatric Medicine.

[B19] Shannon RP, Wei JY, Rosa RM (1986). The effect of age and sodium depletion on cardiovascular response to orthostasis. Hypertension.

[B20] Newman DL, Lallewood RC (1978). The effect of age on the distensibility of the abdominal aorta of man. Surg Gynecol Obstet.

[B21] Josephson RA, Lakatto EG, Katlic MR (1990). Cardiovascular changes in the elderly. Geriatric Surgery.

[B22] Siegel JH, Meakins JL, McClaran JC (1988). The heart and its function in the aged. Surgical care of Elderly.

[B23] Goldman L, Caldera DL, Nussbaum SR (1977). Multi-factorial index of cardiac risk in non cardiac surgical procedures. N Engl J Med.

[B24] Goldman L, Caldera D, Southwick F (1978). Cardiac risk factors and complications in non-cardiac surgery. Medicine.

[B25] Gerson MC, Hurst JM, Hertzberg VS, Baughman R, Rouan GW, Ellis K (1990). Prediction of cardiac and pulmonary complications related to elective abdominal and non-cardiac thoracic surgery in geriatric patients. Am J Med.

[B26] Johnson BD, Evans JG, Williams TF, Beattie BL, Michel JP, Wilcock GK (2000). Age associated changes in pulmonary reserve. Oxford Textbook of Geriatric Medicine.

[B27] Turunen MJ, Peltokallio P (1983). Surgical results in 657 patients with colorectal cancer. Dis Colon Rectum.

[B28] Dhar S, Shastri SR, Lenora RAK (1976). Ageing and respiratory system. Med Clin North Am.

[B29] Wahba WM (1983). The influence of ageing on lung function – clinical significance of changes from age 20. Anaesth Analg.

[B30] Tockman MS, Katlic MR (1990). Aging of the respiratory system. Geriatric Surgery.

[B31] Shea RA, Brooks JA, Dayhoff NE, Keck J (2002). Pain intensity and postoperative pulmonary complications among the elderly after abdominal surgery. Heart Lung.

[B32] McLesky CH, Barash PG, Cullen BF, Stoelting RK (1992). Anaesthesia for the geriatric patient. Clinical Anaesthesia.

[B33] Rupp SM, Castagnoli KP, Fisher DM (1987). Pancuronium and Vencuronium pharmacokinetics and pharmacodynamics in younger and elderly adults. Anaesthesiology.

[B34] Matteo RS, Backus WW, McDaniel DD (1995). Pharmacokinetics and Pharmacodynamics of D-tubocurarie and metacurine in the elderly. Anesth Analg.

[B35] Buxbaum JL, Schwartz AJ (1994). Perianaesthetic consideration for the elderly patient. Surg Clin North Am.

[B36] Zawada ET, Horning JR, Salem AG, Katlic MR (1990). Renal fluid electrolyte and acid base problems during surgery in the elderly. Geriatric Surgery.

[B37] Knight EL, Minaker LM, Evans JG, Williams TF, Beattie BL, Michel JP, Wilcock GK (2000). Disorders of fluid and electrolyte balance. Oxford Textbook of Geriatric Medicine.

[B38] Feest T, Evans JG, Williams TF, Beattie BL, Michel JP, Wilcock GK (2000). Renal Disease. Oxford Textbook of Geriatric Medicine.

[B39] Koruda MJ, Sheldon GF (1991). Surgery in the aged. Adv Surg.

[B40] Shamburek RD, Scott RB, Farrar JT, Katlic MR (1990). Gastrointestinal and liver changes in the elderly. Geriatric surgery.

[B41] Gosain A, DiPietro LA (2004). Ageing and wound healing. World J Surgery.

[B42] Thomas DR (2001). Age-related changes in wound healing. Drugs Ageing.

[B43] Repetto L, Fratino L, Audisio RA, Venturino A, Gianni W, Vercelli M, Parodi S, Dal Lago D, Gioia F, Monfardini S, Aapro MS, Serraino D, Zagonel V (2002). The Comprehensive Geriatric Assessment adds information to ECOG Performance Status in elderly cancer patients:. JCO.

[B44] Folstein MF, Folstein SE, McHugh PR (1975). Mini Mental State: A practical method for grading the cognitive state of patients for the clinicians. J Psychiatatr Res.

[B45] Katz S, Akpom CA (1976). A measure of primary sociological function. Int J Health Serv.

[B46] Lawton MP, Brody EM (1969). Assessment of older people: self-maintaining and instrumental activities of daily living. Gerontologist.

[B47] Brink TL, Yesavage JA, Lum O, Heresuma PH, Adey M, Rose TL (1982). Screening test for geriatric depression. Clin Gerontol.

[B48] Mendoza TR, Wang XS, Cleeland CS, Morrissey M, Johnson BA, Wendt JK, Huber SL (1999). The rapid assessment of fatigue severity in cancer patients: Use of the Brief Fatigue Inventory. Cancer.

[B49] Balducci L, Barnabei R, Albrand G Consensus on Comprehensive Geriatric Assessment. 6^th ^Int. Conf. On Geriatric Oncology: Lyon.

[B50] Welch CS (1948). Surgery in the aged. N Engl J Medicine.

[B51] Repetto L, Granetto C, Venturio A, Balducci L, Lyman GH, Ershler WB (1998). Prognostic evaluation of the older cancer patient. Comprehensive Geriatric Oncology.

[B52] Ferlay J, Bray F, Sankila R, Parkin DM, EUCAN (1999). Cancer Incidence, Mortality and Prevalence in the European Union. IARC Cancer Base No2.

[B53] Vercelli M, Quaglia A, Casella C, Parodi S, Capocaccia R, Martinez Garcia C (1998). Relative survival in elderly cancer patients in Europe. Eur J Cancer.

[B54] Audisio RA, Gennari R, Sunouchi K, Pope D Preoperative Assessment of Cancer in the Elderly (PACE): a pilot study. 94th Annual Meeting AACR.

[B55] (1963). New Classification of Physical status. American Society of Anaesthesiologists. Anaesthesiology.

[B56] Leung JM, Dzankic S (2001). Relative importance of preoperative health status versus intra-operative factors in predicting postoperative adverse outcomes in geriatric surgical patients. L Am Geriatr Soc.

[B57] Michaels JA, Payne SPK, Galland RB (1996). A study of methods used for cardiac risk assessment prior to major vascular surgery. Eur J Vasc Endovasc Surg.

[B58] Knaus WA, Draper EA, Wagner DP, Zimmerman JE (1985). APACHE-ii: a Severity of disease classification system. Crit Care Med.

[B59] Prytherch DR, Whitley MS, Higgns B (1998). POSSUM and Portsmouth POSSUM for predicting mortality. Physiological and Operative Severity Score for EnUmeration of Mortality and Morbidity. Br J Surg.

[B60] Stuck AE, Siu AL, Wieland GD, Adams J, Rubenstein LZ (1993). Comprehensive geriatric assessment: a meta analysis of controlled trials. Lancet.

[B61] Bernabei R, Landi F, Gambassi G, Sgadari A, Zuccala G, Mor V, Rubenstein LZ, Carbonin P (1998). Randomised trial of impact of model of integrated care and case management for older people living in community. Br Med J.

[B62] National Institutes of Health consensus Development Conference statement (1988). Geriatric assessment methods for clinical decision making. J Am Geriatr Soc.

[B63] Rich MW, Beckham W, Wittenberg C, Leven CL, Freedland KE, Carney RM (1995). Multidisciplinary intervention to prevent readmission of elderly patients with congestive heart failure. New Engl J Med.

[B64] Riccardo AA, Roberto G, Koki S, Harikrishnan RN, Ann S, Daniel P, Christ W (2003). Preoperative Assessment of Cancer in Elderly Patients: A Pilot Study. Supportive Cancer Therapy.

[B65] Trimble EL, Carter CL, Cain D, Freidlin B, Ungerleider RS, Friedman MA (1994). Representation of older patients in cancer treatment trials:. Cancer.

[B66] Alberg AJ, Singh S (2001). Epidemiology of breast cancer in older women: Implications for future health care. Drugs & Aging.

[B67] Extermann M (2004). Management issues for elderly patients with breast cancer. Curr Treat Options Oncol.

[B68] Levi F, Lucchini F, Negri E, Boyle P, La-Vecchia C (2001). Changed trends of cancer mortality in the elderly. Ann Oncol.

[B69] Robertson JFR, Todd JH, Ellis IO, Elston CW, Blamey RW (1988). Comparison of mastectomy with tamoxifen for treating elderly patients with operable breast cancer. BMJ.

[B70] Wyld L, Reed MW (2003). The need for targeted research into breast cancer in the elderly. Br J Surg.

[B71] Kenny FS, Robertson JFR, Ellis IO, Elston CW, Blamey RW (1998). Long-term follow-up of elderly patients randomised to primary tamoxifen or wedge mastectomy as initial therapy for operable breast cancer. Breast.

[B72] Precere PE, Wood RAB, Mackie CR, Cushieri A (1982). Tamoxifen as initial sole treatment of localised breast cancer in elderly women: a Pilot study. BMJ.

[B73] Grube BJ, Hamsen NM, Ye W, Herlong T, Guiliano AE (2001). Surgical management of breast cancer in the elderly patient. Am J Surg.

[B74] Diab SAG, Elledge RM, Clark GM (2000). Tumor characteristics and clinical outcome of elderly women with breast cancer. J Natl Cancer Inst.

[B75] Gajdos C, Tartter PL, Bleiweiss IJ, Lopchinsky RA, Bernstein JL (2001). The consequences of under-treating breast cancer in the elderly. J Am Coll Surg.

[B76] Robertson JFR, Ellis IO, Elston CW, Blamey RW (1992). Mastectomy or tamoxifen as the initial therapy for operable breast cancer in elderly patients: 5 year follow up. Eur J Cancer.

[B77] Bates T, Riley DL, Houghton J, Fallowfield L, Baum M (1991). Breast cancer in elderly women: a cancer research campaign trial comparing treatment with tamoxifen and optimal surgery with tamoxifen alone. The elderly Breast cancer working Party:. Br J Surg.

[B78] Gazet JC, Markopoulos C, Ford HT, Coombes RC, Bland JM, Dixon RC (1988). Prospective randomised trial of tamoxifen versus surgery in elderly patients with breast cancer. Lancet.

[B79] Jubelirer SJ, Larzo CR (1998). The treatment of breast cancer in the elderly: a community hospital experience. W V Med J.

[B80] Cerrotta A, Lozza L, Kenda R, Gardani G, Galante E, Zucali R (1997). Current controversies in the therapeutic approach to early breast cancer in the elderly. Rays.

[B81] Nemoto T, Patel JK, Rosner D, Dao TL, Schuh M, Penetrante R (1991). Factors affecting recurrence in lumpectomy without irradiation for breast cancer. Cancer.

[B82] Kesseler HJ, Seton JZ (1978). The treatment of operable breast cancer in the elderly female. Am J Surg.

[B83] Hunt KE, Fry DE, Bland KI (1980). Breast carcinoma in the elderly patient: an assessment of operative risk, morbidity and mortality. Am J Surg.

[B84] Reed MWR, Morrison JM (1989). Wide local excision as the sole primary treatment in elderly patients with carcinoma of the breast. Br J Surg.

[B85] De Haes J-C-J-M, Curran D, Aaronson NK, Fentiman IS (2003). Quality of life in breast cancer patients aged over 70 years, participating in the EORTC 10850 randomised clinical trial. Eur J Cancer.

[B86] Kuehn T, Klauss W, Darsow M, Regele S, Flock F, Maiterth Dahlbender R, Wendt I, Kreienberg R (2000). Long term morbidity following axillary surgery dissection in breast cancer patients – clinical assessment, significance for life quality and impact of demographic, oncologic and therapeutic factors. Breast Cancer Res Treat.

[B87] Kissin MW, Querci della Rovere G, Easton D, Westbury G (1986). Risk of lymphoedema following the treatment of breast cancer. Br J Surg.

[B88] Yancik R, Ries LG, Yates JW (1989). Breast cancer in ageing women. A population- based study of contrasts in stage, surgery and survival. Cancer.

[B89] Chetty U, Jack W, Prescott RJ, Tayler C, Roger A (2000). Management of axilla in operable breast cancer treated by breast conservation: A randomised trial. Edinburgh Breast Unit. Br J Surg.

[B90] Gaskell DJ, Hawkins RA, De Carteret S, Chetty U, Sangster K, Forrest APM (1992). Indications for primary tamoxifen therapy in the elderly women with breast cancer. Br J Surg.

[B91] Wilsher PC, Robertson JFR, Jackson L, Al Hilaly M, Blamey RW (1997). Investigation of primary tamoxifen therapy for elderly patients with operable breast cancer. Breast.

[B92] Low SC, Dixon AR, Bell J, Ellis IO, Elston CW, Robertson JFR, Blamey RW (1992). Tumour oestrogen receptor content allows selection of elderly patients with breast cancer for conservative tamoxifen treatment. Br J Surg.

[B93] Fenessy M, Bates T, MacRae K, Riley D, Houghton J, Baum M (2004). Late follow-up of a randomized trial of surgery plus tamoxifen versus tamoxifen alone in the women aged over 70 years with operable breast cancer. Br J Surg.

[B94] Lipa JE, Youssef AA, Kuerer HM, Robb GL, Chang DW (2003). Breast recosntruction in older women: Advantages of auto-genous tissue. Plast Reconstr Surg.

[B95] Audisio RA, Osman N, Audisio MM, Montalto F (2004). How do we manage breast cancer in the elderly: a survey among members of the British Association of Surgical Oncologists (BASO). Crit Rev Oncol Hematol.

[B96] Chang CK, Ira A, Jacobs, Vida M, Vizgirda RN, George IS (2003). Melanoma in the Elderly patient. Arch Surg.

[B97] Austin PF, Cruse CW, Lyman G, Schroer K, Glass F, Reintgen DS (1994). Age as a prognostic factor in the malignant melanoma population. Ann Surg Oncol.

[B98] Cohen HJ, Manton K, Woodbury M (1987). Malignant Melanoma in the Elderly. J clin oncol.

[B99] Chang CK, Jacobs IA, Theodosiou E, Salti GI (2003). Thick Melanoma in the elderly. American Surgeon.

[B100] Nooda EM, Vrouenraets BC, Nieweg OE, Van Geel AN, Eggermont AMM, Kroon BBR (2002). Safety and efficacy of isolated limb perfusion in elderly melanoma patients. Ann Oncol.

[B101] Geller AC, Sober AJ, Zhang Zi, Brooks DR, Miller DR, Halpern A, Gilchrest BA (2002). Strategies for improving melanoma education and screening for men ≥ 50 years: Findings from the American Academy of Dermatological National Skin Cancer Screening Program. Cancer.

[B102] Cangemi V, Volpino P, D'Andrea N, Puopolo M, Tomassini R, Cangemi R, Piat G (1996). Lung cancer surgery in elderly patients. Tumori.

[B103] Levi F, La Vecchia C, Lucchini F, Negri E (1996). Worldwide trends in cancer mortality in the elderly-1955–1992. Eur J Cancer.

[B104] US Census Bureau (2000). International Database.

[B105] Levi F, Lucchini F, Negri E, Boyle P, La-Vecchia C (2001). Changed trends of cancer mortality in the elderly. Ann Oncol.

[B106] Zanetti R, Crosignani P (1992). Cancer in Italy – Incidence data from Cancer Registries 1983–1987. Torino.

[B107] National Cancer Institute (2000). SEER Cancer Statistics Review 1973–1997.

[B108] Anderson R (1999). United States Life Tables, 1997. National Vital Statistics. Reports.

[B109] Damhuis RA, Schutte PR (1996). Resection rates and postoperative mortality in 7,899 patients with lung cancer. Eur Respir J.

[B110] Teeter SM, Holmes FF, McFarlane MJ (1987). Lung carcinoma in the elderly population. Influence of histology on the inverse relationship of stage to age. Cancer.

[B111] Miller JI, Hatcher CR (1987). Limited resection of bronchogenic carcinoma in the patient with marked impairment of pulmonary function. Ann Thorac Surg.

[B112] Pastorino U, Valente M, Bedini V, Infante M, Tavecchio L, Ravasi G (1991). Limited resection for Stage I lung cancer. Eur J Surg Oncol.

[B113] Jaklitsch MT, Mery CM, Bueno R, Vasconcelles MJ, Richards WG, Mentzer S, DeCamp M, Swanson S, Lukanich J, Sugarbaker D (1999). Lesser pulmonary resections are more common in elderly non-small cell lung cancer (NSCLC) patients but do not adversely affect survival. Proc Am Soc Clin Oncol.

[B114] Jaklitsch MT, Pappas EA, Bueno R (2004). Thoracoscopic Surgery in Elderly Lung Cancer patients. Critical reviews in Oncology / haematology.

[B115] Okada S, Ishimori S, Sato M, Sato S (2001). Thoracoscopic lung resection from a peripheral lung cancer by a single surgeon with a voice controlled robot. Kyobu Geka Oct.

[B116] Kirby TJ, Mack MJ, Landreneau RJ, Rice TW (1995). Lobectomy – video-assisted thoracic surgery versus muscle-sparing thoracotomy: A randomized trial. J Thorac Cardiovasc Surg.

[B117] McKenna RJ, Wolf RK, Brenner M, Fischel RJ, Wurnig P (1998). Is lobectomy by video-assisted thoracic surgery an adequate cancer operation?. Ann Thorac Surg.

[B118] Jaklitsch MT, Mery CM, Audisio RA (2003). The use of surgery to treat lung cancer in elderly patients. The Lancet Oncol.

[B119] Roth JA, Fossella F, Komaki R, Ryan MB, Putnam JB, Lee JS, Dhingra H, De Caro L, Chasen M, McGavran M (1994). A randomized trial comparing Perioperative chemotherapy and surgery with surgery alone in resectable stage IIIA non-small-cell lung cancer. J Natl Cancer Inst.

[B120] Thomas P, Doddoli C, Neville P, Pons J, Lienne P, Giudicelli R, Giovannini M, Seitz JF, Fuentes P (1996). Esophageal cancer in elderly. Eur J Cardiothorac Surg.

[B121] Peracchia A, Bardini R, Ruol A (1988). Carcinoma of esophagus in elderly (70 years of age or older): Indications and results of surgery:. Dis of Esophagus.

[B122] Gorman RC, Morris JB, Kaiser LR (1994). Esophageal disease in the elderly patient. Surg Clin North Am.

[B123] Ellis FH, Williamson WA, Heatley GJ (1998). Cancer of Esophagus and cardia: Does Age influence treatment selection and surgical outcomes?. J Am Coll Surg.

[B124] Thomas P, Doddoli C, Neville P, Pons J, Lienne P, Giudicelli R, Giovannini M, Seitz JF, Fuentes P (1996). Esopahgeal cancer resection in the Elderly. Eur J Cardiothora Surg.

[B125] Alexiou C, Beggs D, Salama FD, Brackenbury ET, Morgan WE (1998). Surgery for oesophgeal cancer in elderly patients: view from Nottingham. J Thorac Cardiovasc Surg.

[B126] Sharpe DA, Moghissi K (1996). Resectional surgery in carcionoma of esophagus and cardia: what influences long-term survival?. Eur J Cardiothorac Surg.

[B127] Young JL, Percy CL, Asire AJ, (eds) (1981). Surveillance, Epidemiology, and End results: Incidence and mortality data 1973–77.

[B128] Hirano T, Yamamoto S, Miura T (1990). Cancer of the stomach in the elderly. Res Surg.

[B129] Rohde H, Bauer P, Stutzer H, Heitmann K, Gebbensleben B (1991). Proximal compared with distal adenocarcinoma of the stomach: differences and consequences. Br J Surg.

[B130] Tamura P, Curtiss C (1960). Carcinoma of the stomach in the young adult. Cancer.

[B131] Matsushita I, Hanai H, Kajimura M, Tamakoshi K, Nakajima T, Matsubayashi Y, Kanek E (2002). Should gastric cancer patients more than 80 years of age undergo surgery? Comparison with patients not treated surgically concerning prognosis and quality of life. J Clin Gastroenterol.

[B132] Saidi RF, Bell JL, Dudrick PS (2004). Surgical resection of gastric cancer in elderly patients: Is there a difference in outcome. J Surg Res.

[B133] Maehara Y, Oshiro T, Oiwa HH, Oda S, Baba H, Akazawa K, Sugimachi K (1995). Gastric Carcinoma in patients' over 70 years of age. Br J Surg.

[B134] Wald A, Raymond T, Hower F, Brocklehurst JC (1999). Large Bowel. Brocklehurst's Textbook of Geriatric Medicine and Gerontology.

[B135] Seidfeldin R, Hantsch JJ (1999). The economic burden associated with colon cancer in the United states. Clin Ther.

[B136] Young JL, Percy CL, Asire AJ (1981). Surveillance, Epidemiology, and End results: Incidence and mortality data, 1973–77.

[B137] Tekkis PO, Poloniecki JD, Thompson MR, Stamatakis JD ACPGBI Colorectal cancer study 2002 – Part A: Unadjusted outcomes.

[B138] Capra E, Scintu F, Zorco L, Casula G (2003). Surgical treatment of colorectal cancer in patients over 80 years. Short and long term results. Minerva Chirur.

[B139] Koperna T, Kisser M, Schulz F (1997). Emergency surgery for colon cancer in the aged. Arch Surg.

[B140] Ficorella C, Cannita K, Ricevuto E (1999). The adjuvant therapy of colonic carcinoma in old age. Minerva Med.

[B141] Jaana VH, Peter S, Antero HI, Ilmo KH (2004). Complications and Survival after surgery for rectal cancer in patients younger than and aged 75 years or older. Dis Colon Rec.

[B142] Jane AH, Karen EM, Anthony LAF (2003). Systematic review of management of colorectal cancer in elderly patients. Clin Colorectal Cancer.

[B143] Kapiteijn E, Van de Velde CJ (2002). The role of total mesorectal excision in the management of rectal cancer. Surg Clin North Am.

[B144] Ho P, Law WL, Chan SC, Lam CK, Chu KW (2003). Functional outcome following low anterior resection with total mesorectal excision in the elderly. Int J Colorectal Dis.

[B145] Philip PS, Farquharson SM, Sexton R, Heald RJ, Moran BJ (2004). Rectal cancer in the elderly: Patient's perceptions of bowel control after restorative surgery. Dis Colon Rectum.

[B146] Brand MI, Saclarides TJ, Dobson HD, Millikan KW (2000). Liver resection for colorectal cancer: Liver metastases in the aged. Am Surg.

[B147] Audisio RA, Cazzaniga M, Veronesi P, Andreoni B, Aapro MS (1997). Elective Surgery for colorectal cancer in the aged; A clinico- economic evaluation. Br J Cancer.

[B148] Patankar SK, Larach SW, Ferrara A, Willaimson PR, Gallagher JT, De Jesus S, Narayanan S (2003). Prospective Comparison of laparoscopic Vs open resections for colorectal adenocarcinoma over a ten year period. Dis Colon Rectum.

[B149] Nelson H, Sargent DJ, Wieand HS, Fleshman J, Anvari M, Stryker SJ, Beart RW, Hellinger M, Flanagan R, Peters W, Ota D (2004). A Comparison of Laparoscopically Assisted and Open Colectomy for Colon Cancer. N Engl J Med.

[B150] Gonzalez QH, Heslin MJ, Shore G, Vickers SM, Urist MM, Bland KI (2003). Results of long-term follow-up for transanal excision for rectal cancer. Am Surg.

[B151] De Graaf EJ, Doornebosch PG, Stassen LP, Debets JM, Tetteroo GW, Hop WC (2002). Transanal endoscopic microsurgery for rectal cancer. Eur J Cancer.

[B152] Azimuddin K, Riether RD, Stasik JJ, Rosen L, Khubchandani IT, Reed JF (2000). Transanal endoscopic microsurgery for excision of rectal lesions: technique and initial results. Surg Laparosc Endosc Percut Tech.

[B153] Neary P, Makin GB, White TJ, Hartley J, MacDonald A, Lee PW, Monson JR (2003). Transanal endoscopic microsurgery: a viable operative alternative in selected patients with rectal lesions. Ann Surg Oncol.

